# A Ghostly Warning

**Published:** 1892-02

**Authors:** 


### A GHOSTLY WARNING.

No, I don't believe in ghosts, said a locomotive engineer on one of the trunk lines running out of Kansas City; most of the phenomena we run across can be explained on some better hypothesis, and of those that can't the majority are due to illness. Yes, I have had some experiences myself that I could not explain at the time, and have never since been able to, but I still believe they must have some rational explanation. One of the strangest incidents in my life occurred when I was running an engine on the Chicago and Alton, from Slater to Roodhouse. You know there is a little station near Centralia, Mo., called Clark, where the Chicago and Alton and the Wabash cross. At this crossing we always have to come to a full stop, and, though I have gone over it a thousand times, I never go over it without a shudder. Whenever I take an engine over that crossing a lady's voice as gentle and as clear as a silver bell, always says to me plainly and distinctly: "All right; go ahead." There have been only three times that this has not been done. Once in 1883, she said to me:

"Stop! wait a minute!"

So accustomed had I become to obeying her that I stopped at once at the unusual command. Looking ahead I saw by my headlight that a man had caught his foot in the frog just ahead, and would have been crushed if I had gone ahead. He himself said it was a d——d close call, but on learning that he was the editor of one of the little country weeklies of Missouri, I didn't tell him why I had stopped, for I had no desire to have it published, my "queer" imagination having already caused unfortunate talk.

After that, for a year, everything went as usual, and I always heard the same gentle voice say :

"All right ; go ahead !"

One day after I had come to a dead stand still, and had started up again, the lady's voice said to me, in quick, anxious tones :

"Stop ! Wait a minute ! You'll be too late ! Reverse !"

I followed her command as quickly as possible, and a little golden haired fairy was picked up from just in front of the wheels of the locomotive. The mother, who was waiting for a Wabash train fainted, and was still unconscious when we went on, but she afterwards sent a handsome check to the division superintendent for me at Slater. Now, the strangest part of all this is the fact that no one ever heard these commands of warning except me. When we had saved this little girl I mention, the fireman asked me if I saw the child and I at once said "No," and of course, I then had to explain why I had stopped and reversed, and this revived the talk which had been very common a year or more before with regard to my "queer" ways, but which had been dropped. When the superintendent later sent for me to give me the check that had been sent, he asked me the facts and I told him the whole story just as it really existed. He looked at me a little queerly when I got through, and said :

"You have been too long with us, and are too serviceable for us to want to lose you, but we can't have any stories like this circulating. It will injure our service too badly." I suggested that only twice had it interfered with my run for a half minute, and in both cases had saved a life, but this did no good, and I saw that a repetition of yielding to my imagination as he called it, would probably mean my resignation. It happened that I was requested to take the place of a friend and run back over the road that very night, and although I had lost a great deal of sleep, I couldn't refuse. When we approached the Clark switch and crossing I was very uneasy and nervous and felt that my imagination was just in condition to serve me any sort of a trick.

We stopped, and as I started up again I heard that voice I knew so well say, with perfect distinctness :

"Stop ! Wait a minute !"

Now, I thought to myself, I must not yield to my imagination, like a child. So I made no movement to stop.

She spoke again, and said in agony, as it seemed :

"For heaven's sake stop! Reverse!" But instead of doing so, I gave her more steam and as we went forward I imagined I heard the engine crying something, and I know I heard the warning voice in tears, crying: "Oh, dear! Oh, dear!" When I came back on my return I learned that our train had run over an old man, who lived a few minutes, but was never identified. I went over the run once or twice afterwards, but that heartrending cry of "Oh, dear! Oh, dear!" rings in my ear every time I go past Clark, and so I told the superintendent and handed in my resignation.

"And yet you don't believe in ghosts?" said a listener.

"No," he replied. "Of course I can't explain this, but it must have some reasonable explanation."

